# Effectiveness of functional electrical stimulation machine in managing neurological diseases - A retrospective study

**DOI:** 10.12669/pjms.40.2(ICON).8966

**Published:** 2024-01

**Authors:** Farah Naz, Danish Hussain, Hussain Ali, Qasim Raza, Farrukh Siddique

**Affiliations:** 1 Farah Naz, Department of Physical Therapy, Indus Hospital and Health Network, Karachi, Pakistan; 2 Danish Hussain, Department of Physical Therapy, Indus Hospital and Health Network, Karachi, Pakistan; 3 Hussain Ali, Department of Physical Therapy, Indus Hospital and Health Network, Karachi, Pakistan; 4 Qasim Raza, Department of Physical Therapy, Indus Hospital and Health Network, Karachi, Pakistan; 5 Farrukh Siddique, Department of Physical Therapy, Indus Hospital and Health Network, Karachi, Pakistan

**Keywords:** Functional Electrical Stimulation, neurological diseases, Cardio vascular accident (CV), Stroke, Manual Muscle Testing

## Abstract

**Background & Objective:**

Cerebrovascular Accident (CVA) or stroke, Spinal Cord injury can lead to neurological diseases resulting in major loss in motor function causing hemiplegia or tetraplegia. In 2019, according to The Global Burden of Diseases (GBD) CVA/Stroke is the second leading cause of death and the third leading cause of death and disability combined, globally. Its prevalence vary drastically among South Asian countries. The objective of this study was to determine the effectiveness of Functional Electrical Stimulation (FES) machine on neurologically impaired patients at the Physical Therapy department at IHHN, Karachi, Pakistan.

**Method:**

In this retrospective study data was extracted from August 2016 to February 2022 on patients with neurological symptoms i.e. hemiplegia or paraplegia with muscle power of two or less on Manual Muscle Testing (MMT). The parameters for evaluating patients progress pre and post treatment were MMT results and their mobility status. The number of sessions ranged between 40 to 100 sessions of Functional Electrical Stimulation (FES) provided on alternate days according to the patient’s need.

**Result:**

Data of 51 patients who had completed their treatment were extracted and analyzed. The mean age of patients who completed treatment was 49.62 ± 17.26 years. Out of 51(100%), 30 (58.8%) were male and 21 (41.2%) were female. Pre- and post-treatment median (IQR) showed remarkable improvement in MMT of upper limb muscle (from 1.0 to 4.0) and lower limb muscle (from 2.0 to 4.0).

**Conclusion:**

FES cycling is an effective treatment for patients with neurological impairments, as it resulted in improvement in both upper and lower limb muscle strength, along with mobility status.

## INTRODUCTION

Neurological diseases lead to significant motor and sensory function deficits, including hemiplegia or tetraplegia resulting from conditions such as cerebrovascular accident (CVA) or stroke, spinal cord injury (SCI), and other neurological disease.^1^

Cerebrovascular accident or stroke, occurs when there is blockage or rupture in an artery leading to disruption of oxygen supply i.e., ischemia. This disruption results in the sudden death of specific brain cells due to sustained oxygen deprivation.^2^,^3^ Ischemic stroke due to artery occlusion is a prevalent global cause of disability and mortality.^4^ Annually, 15 million strokes occur, resulting in one-third mortality and one third permanent disability, significantly impacting patients, families and communities.^4^

Spinal cord injury (SCI) refers to cord damage from trauma, possibly causing temporary or complete loss of nerve function,^5^ these are catastrophic events causing physical, emotional, psychological, and financial hardships for patients, their families, and society.^6^

Traumatic Brain Injury (TBI) results from external trauma, altering brain structure and function. Globally, about 50 million people experience traumatic brain injury (TBI) annually, with enduring physical, cognitive, and emotional impacts.^7^,^8^ Studies from the United States and New Zealand report 500-800 new TBI cases annually per 100,000 individuals.^9^

In 2006, the World Health Organization (WHO) identified neurological diseases as the leading health concern, comprising 6.3% of the total disability-adjusted life-years (DALYs) burden.^10^ In 2019, The Global Burden of Diseases (GBD) described stroke as second-leading cause of death and the third-leading cause of death and disability combined in the world.^11^,^12^ A study by Kang et al. revealed 821.8 million new neurological disorder cases worldwide, with 227 million in the WHO South-East Asian region and 178.6 million in the WHO Western Pacific region.^13^ And according to Farooq et al study in 2021, the estimated incidence of stroke in Pakistan is 250/100,000 annually.^14^

Functional Electrical Stimulation (FES) bike is multichannel electrical stimulation with functional movement used for enhancing impaired motor function by electrically stimulating skeletal muscles in patients experiencing neurological deficits.^15^,^16^ FES promotes active, repetitive movement in paralyzed limbs, leading to improved function, range of motion (ROM), increased muscle strength, and enhanced muscle tone in individuals with neurological conditions like stroke and spinal cord injury.^15^,^16^ The FES bike consists of an electrical stimulator, stimulation electrodes, upper and lower limb ergometer, and a display monitor. Medical Research Council Manual Muscle Testing (MMT) is a scale to measure muscle strength, scored 0 to five points.^17^

## METHODS

A retrospective study was conducted at Indus Hospital & Health Network (IHHN), Karachi, on patients with limb paralysis due to neurological damage. Electronic medical records were extracted from August 2016 to February 2022 of patients who had completed treatment.

### Ethical Approval

Research proposal was approved by IHHN, Institutional Review Board (IRB) with study approval, IHHN_IRB-2023_04_004.

### Inclusion Criteria

Males and females, aged 13 to 87 years, who presented with hemiplegia or diaplegia due to Cerebrovascular Accident (CVA), Spinal Cord Injury (SCI), Guillain-Barre Syndrome (GBS), or Transverse Myelitis with intact cognitive drive, muscle power on MMT less than and equal to two were included in the study. Intact cognitive function was necessary for the FES intervention to avoid injury.

### Exclusion Criteria

Patients with physical deformities (unable to perform FES intervention) and patients who had not completed treatment or had incomplete records were excluded.

### Data collection

The 51 electronic medical records which met inclusion and exclusion criteria were used to extract data on muscle power of paralyzed limb i.e. the affected side’s upper and lower limb (shoulder flexor, elbow flexors, elbow extensor, quadriceps, hamstring, tibialis anterior, and gastrocnemius) at baseline; and after a minimum of 40 to a maximum of 100 sessions (3 sessions every alternate day per week) depending on the patient’s need. Mobility status (wheelchair-dependent, walker-assisted, elbow stick-assisted, support-assisted, or independent walking) was recorded at baseline and post-treatment.

### Statistical Analysis

Data of completed patients were analyzed using software SPSS 26. Mean ± SD for age and median (IQR) of MMT of shoulder flexors, elbow flexors, elbow extensors, quadriceps, hamstring gastrocnemius, tibialis anterior was calculated based on the normality of data. Frequency and percentages of gender, residency of patients, transportation mode (using Indus van or other modes) and diagnosis was calculated. Wilcoxon test was applied to compare pre and post improvement in MMT of upper and lower limb based on normality of data (Sphiro-Wilk test used to check normality). McNemar-Bowker Test applied to compare pre and post mobility status, p-value <0.001 was considered statistically significant.

## RESULTS

Analysis was done on data of 51 patients who completed their FES treatment between August 2016 to February 2022. The mean age calculated was 49.62 ± 17.26 years. Out of 51 patients 30 (58.8%) were male and 21 (41.2%), were female. Forty-two individuals (82.4%) were resident of Korangi district, while nine individuals (17.6%) came from outside Korangi. Thirty-seven patients (72.5%) utilized Indus-provided transportation, while 14 (27.5%) used personal conveyance. Most frequently documented diagnosis was Cerebrovascular Accident (CVA), with 23 cases (45.1%) displaying right-sided weakness, and 19 cases (37.3%) manifesting left-sided weakness. (Table-I)

**Table-I:** Baseline status.

Age (Years) *	
Mean ± Standard Deviation	49.62 ± 17.26
Min – Max	13.00 – 87.00
***Gender***	
Male	30 (58.8%)
Female	21 (41.2%)
***Residency***	
Korangi	42 (82.4%)
Outside Korangi	9 (17.6%)
***Mode of transportation***	
Self	14 (27.5%)
Indus Van	37 (72.5%)
***Patients Diagnosis Neurological Diseases***	
CVA-Left sides weakness	19 (37.3%)
CVA-Right sides weakness	23 (45.1%)
GBS	3 (5.9%)
Nerve Injury	1 (2.0%)
SCI	4 (7.8%)
Transverse Myelitis	1 (2.0%)

* Normality of age was checked by Sphiro-Wilk Test.

Pre and post treatment median (IQR) of MMT of shoulder flexors, elbow extensors, elbow flexors, quadriceps, hamstring, gastrocnemius and tibialis anterior muscle subsequently are shown in Table-II. Pre and post treatment median (IQR) showed remarkable improvement in manual muscle testing (MMT) of upper limb (from 1.00 to 4.00) muscles and lower limb (from 2.00 to 4.00) muscles. P value is <0.0001 depicts that FES has statistically significant effect on neurologically impaired patients who had muscular weakness as shown in Table-II.

**Table-II:** Comparison of Pre and Post treatment MMT.

		Pre	Post	P- value
MMT of Shoulder Flexors	Median (IQR)	1 (0 - 2)	4 (3 - 4)	0.000^**1^
	Min -Max	0 - 3	2 - 5	
MMT of Elbow Flexors	Median (IQR)	1 (0 - 2)	4 (3 - 4)	0.000^**1^
	Min -Max	0 - 3	2 - 5	
MMT of Elbow Extensors	Median (IQR)	1 (0 - 2)	4 (3 - 4)	0.000^**1^
	Min -Max	0 - 3	2 - 5	
MMT of Quadriceps	Median (IQR)	2 ( 0 - 2)	4 (4 - 5)	0.000^**1^
	Min -Max	0 - 3	2 - 5	
MMT of Hamstring	Median (IQR)	2 (0 - 2)	4 (4 - 5)	0.000^**1^
	Min -Max	0 - 3	2 - 5	
MMT of Tibialis Anterior	Median (IQR)	2 (0 – 2)	4(4–5)	0.000^**1^
	Min -Max	0 -3	2 -5	
MMT of Gastrocnemius	Median (IQR)	2 (0– 2)	4 (4–5)	0.000^**1^
	Min -Max	0 -3	2-5	

** P<0.001,

1 Wilcoxon Test.

Results at baseline illustrated that of 32(62.7%) wheel chair dependent patients, 7(13.7) remained on wheel chair after treatment till last follow up, 3(5.9%) walked with walker, 10(19.6%) begun walking with help of a stick, 4(7.8%) started walking with support and 8(15.7%) started walking independently. One patient who was walking with walker was able to walk independently post treatment. Patients 2 (3.9%) using stick for walk began walking independently after treatment. Results showed 27.5% patients who once walked with assistance, after treatment, out of them 10(19.6%) patients could walk independently and 4(7.8%) incapacitated patients started using stick to walk. P value was statistically significant <0.0001, Table-III

**Table-III:** Mobility Status Before and after treatment

	Mobility Status	After Treatment						

		Wheel Chair Dependent	Walking with Walker	Walking with stick	Walking with Someone Support	Walking Independently	Total	P-value
Before Treatment	Wheel Chair Dependent	7(13.7%)	3(5.9%)	10(19.6%)	4(7.8%)	8(15.7%)	32 (62.7%)	0.000^**†^
	Walking with Walker	-	-	-	-	1(2.0%)	1(2.0%)	
	Walking with stick	-	-	-	-	2(3.9%)	2(3.9%)	
	Walking with Someone Support	-	-	4(7.8%)	-	10(19.6%)	14 (27.5%)	
	Walking Independently	-	-	-	-	2 (3.9%)	2 (3.9%)	
	Total	7(13.7%)	3(5.9%)	14(27.5%)	4(7.8%)	23(45.1%)	51(100.0%)	

** P<0.001, † McNemar-Bowker Test.

## DISCUSSION

This study shows a substantial improvement in median (IQR) manual muscle testing (MMT) scores for both upper and lower limb muscles pre- and post-treatment. Notably, patients with neurological diseases experienced a significant improvement in muscle strength, going from an MMT score below 2.00 to 4.00.There are several studies on Functional Electrical Stimulation (FES) which have demonstrated its practicality in addressing dropped foot, leading to increased walking speed.^18^,^19^ reduced walking effort,^20^ decreased incidence of falls,^21^ and a positive impact on daily activities^21^,^22^ as well as quality of life.^23^ Furthermore, this technique has demonstrated a training effect on walking speed in individuals with stroke and non-progressive neurological conditions.^24^

In addition, this study observed improved mobility among patients. Of the initial 32 wheelchair-dependent patients, four started walking independently, 10 could walk with assistance, three used a walking stick, and three transitioned from wheelchairs to walkers. Notably, only seven patients continued to rely on wheelchairs, and importantly, none experienced mobility deterioration. According to a study, patients in the hospital took their first steps after a stroke in an average of 18.1±8.4 days with Functional Electrical Stimulation (FES), compared to 20.2±6.8 days with placebo stimulation and 21.2±8.0 days with standard rehabilitation.^25^ This suggests that individuals undergoing FES treatment typically started walking two to three days earlier than those in the placebo or standard rehabilitation groups. A meta-analysis found that Functional Electrical Stimulation (FES) did not result in improved gait speed compared to conventional treatment.^26^

In the same meta-analysis, a sensitivity analysis showed that combining Functional Electrical Stimulation (FES) with physiotherapy led to improved gait speed compared to physiotherapy alone in a group of 133 individuals, with an effect size of 0.51 (95% CI: 0.16 to 0.86) and no significant heterogeneity (I2 0%, P = 0.0042).^26^ Garzon et al and Calabro et al demonstrated that FES uses electrical impulses to stimulate muscle contractions, improving functional movements and enhancing muscle strength in individuals with neurological conditions like stroke and spinal cord injuries.^12^,^27^ Sbruzzi G et al.^28^ established a direct relationship between stimulation frequency and muscle strength. They observed that higher stimulation frequency led to increased motor unit recruitment and, consequently, greater muscle strength.

Overall, their study emphasized significant muscle strength improvement through FES treatment.^26^ A study describes FES as a therapeutic approach utilizing neuroplasticity to restore voluntary movement after stroke and spinal cord injury.^14^ In a study of 99 subjects with chronic hemiparesis, improved mobility was observed following 30 weeks of FES treatment.^27^ Our study resonating similar findings.

### Limitations

It includes small sample size of 51 participants who completed FES treatment. Due to the absence of a Neurology Department, access to additional demographic variables for a broader perspective was restricted.

## CONCLUSION

Functional Electrical Stimulation cycling effectively enhances muscle strength in both upper and lower limbs, increasing scores from 1.00 to 4.00 and 2.00 to 4.00, respectively, and also improves patient mobility.

### Authors’ Contribution:

**FN** conceived, designed, statistical analysis & is responsible for the integrity of research.

**DH** prepared drafts and review after final approval.

**HA** acquisition of data and manuscript writing.

**QR** did manuscript writing.

**FS** helped in manuscript rewriting and analysis after review.
